# Supplementary material for: Change of human mobility during COVID-19: A United States case study
Source: PLoS One. 2021 Nov 2;16(11):e0259031. doi: 10.1371/journal.pone.0259031 (PMC8562789; doi:10.1371/journal.pone.0259031)
Supplement: S1 File — (PDF) [file pone.0259031.s001.pdf]

Supplement to
"Change of human mobility during COVID-19: A United States case study"

Justin Elarde[1], Joon-Seok Kim [1], Hamdi Kavak[2], Andreas Züfle[1], Taylor Anderson[1*]

[1] Department of Geography and Geoinformation Science
[2] Department of Computational and Data Sciences
George Mason University, Fairfax, VA, USA

[*] Corresponding Author, tander6@gmu.edu

1 Implementation and Source Code

In this study, we used the Python programming language for data collection and analysis. The main Python packages used were Pandas, Matplotlib, Scikit-Learn, and Pysal. Pandas along with Matplotlib allowed for data pre-processing as well as visualization. Scikit-Learn was used for the truncated SVD and the clustering techniques. Finally, Pysal was used for performing both the local and global spatial autocorrelation analysis. All additional packages used in this research and our implementation can be found in our GitHub repository at https://github.com/GMU-GGS-NSF-ABM-Research/Mobility-Trends.

2 Data Set and Data Processing Details

We have made our derived data $\Delta TSPP$ for every county fully available on the GitHub repository. The following list of counties are removed from our analysis as outliers with a standard deviation of four or greater in the PCA space: Grant County, AR, San Mateo County, CA, Santa Clara County, CA, Howard County, MD, Somerset County, NJ, Fairfax County, VA, Loudoun County, VA.

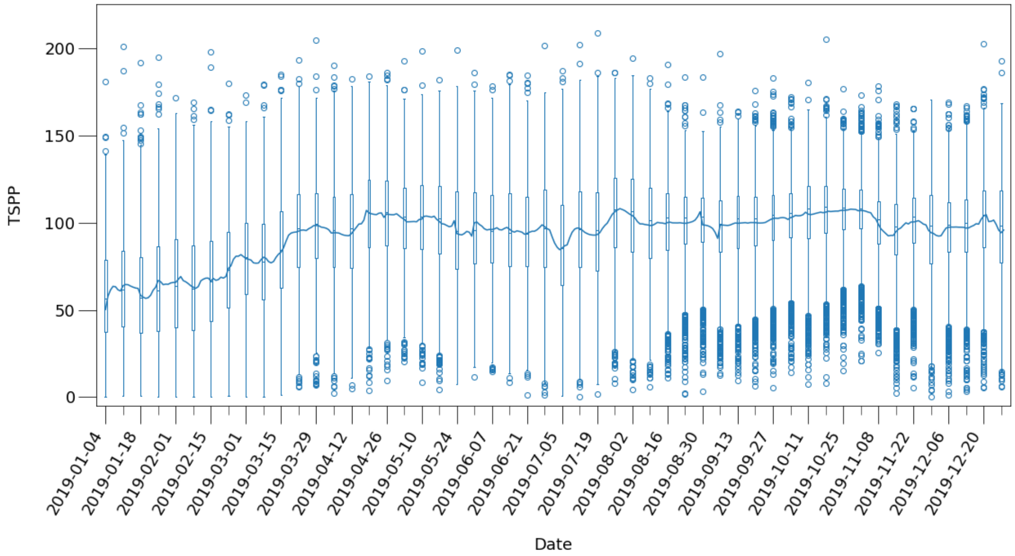

Figure 1: Boxplot for 2019 TSPP.

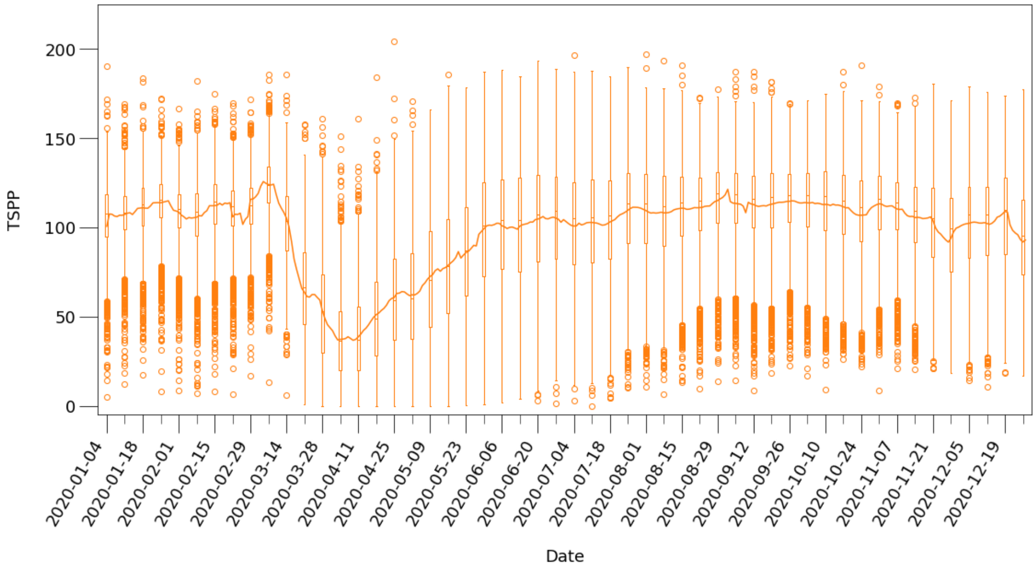

Figure 2: Boxplot for 2020 TSPP.

3 Analysis Details

3.1 TSPP Boxplots

Boxplots describing the weekly distribution of the TSPP data for 2019 (Figure 1) and 2020 (Figure 2) are presented.

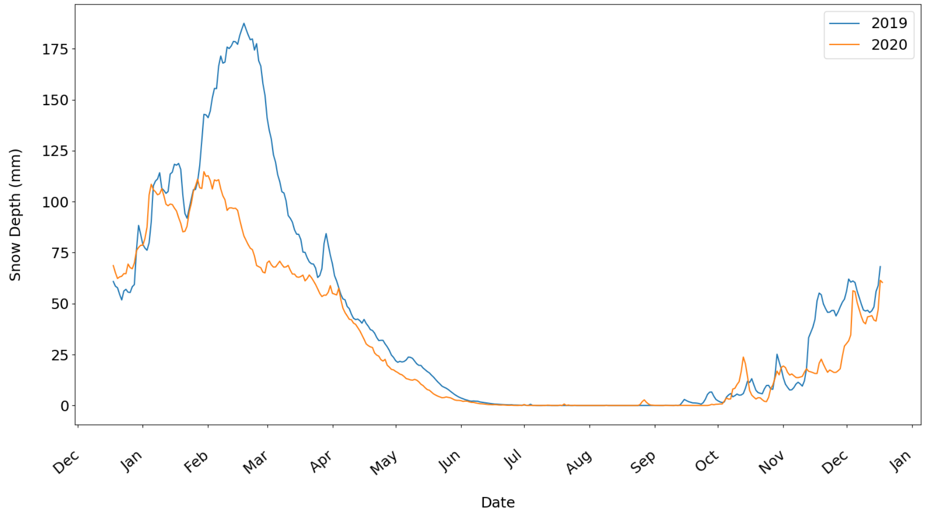

Figure 3: US snow depth (mm) in 2019 and 2020.

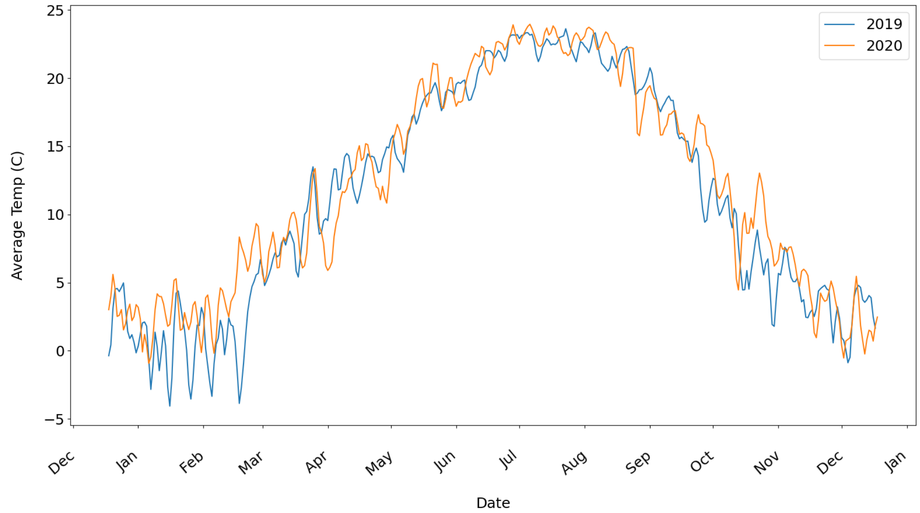

Figure 4: Average US temperature in 2019 and 2020.

3.2 Explanation of Seasonal Mobility using Weather Data

We observe anomalously high mobility in January and February 2020. This is likely a combined effect due to higher-than-average temperatures, below-average snow depth, and panic buying behaviors. Here, Figure 3 and 4 compares the trends in snow depth and average temperature obtained from NOAA's Global Historical Climatology Network, respectively, between 2019 and 2020 across US. These findings support the mobility trends observed in the data.

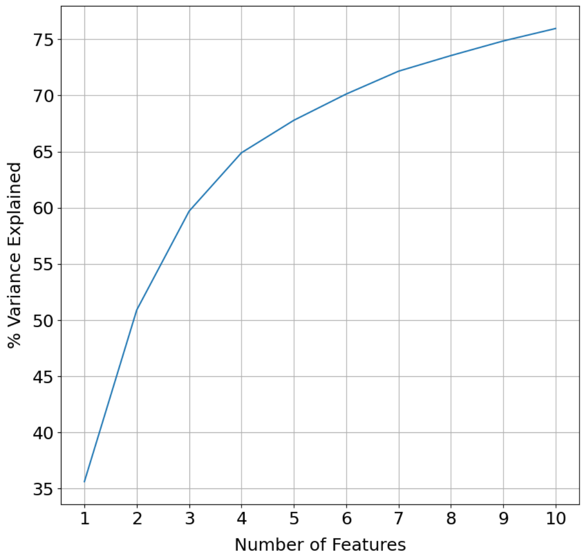

Figure 5: Explained variance for different numbers of latent features.

3.3 Principal Component Analysis

Figure 5 and Table 1 presents the amount of explained variation for each feature in the Principal Component Analysis. Based on the elbow method, we find that three features are sufficient for explaining variation (1).

PC	Variance Explained
PC1	0.36
PC2	0.15
PC3	0.089
PC4	0.049
PC5	0.028
PC6	0.022
PC7	0.019
PC8	0.014
PC9	0.012
PC10	0.011

Table 1: Variance explained for each PC.

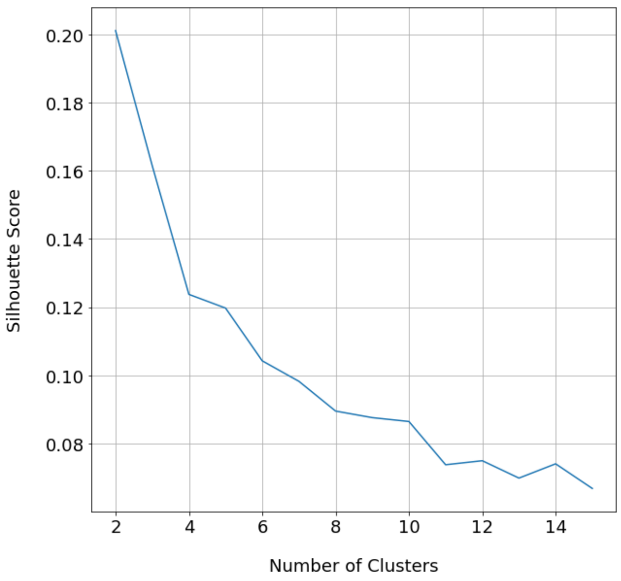

Figure 6: K-means silhouette scores.

3.4 Clustering Analysis

For clustering, we compare between the K-means algorithm (2) and hierarchical clustering algorithm (3). The K-means algorithm partitions n observations into k clusters by randomly initializing k points (means or cluster centroids) and assigning each observation to their closest point. Cluster centroids are updated and iteratively reassigned until convergence. In contrast, either agglomerative or divisive hierarchical clustering does not require the number of clusters to be specified. Based on the distance between features,

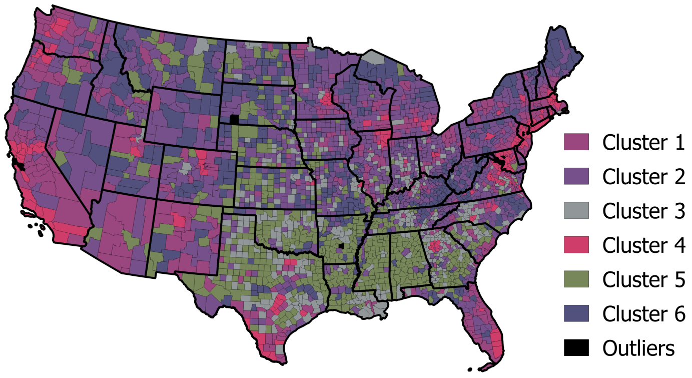

Figure 7: Clustering result where k = 6.

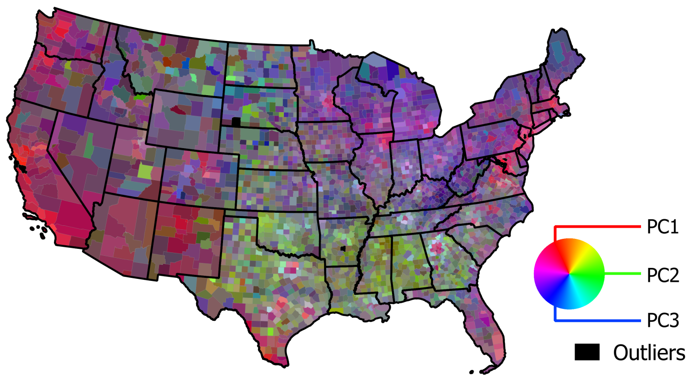

Figure 8: RGB Map of Latent Features of Change of Exposure ΔMoPE for all counties in the US.

a hierarchy of clusters is built from a set of single observations to one single cluster of all observations.

In the case of hierarchical clustering, we tested different distance functions, including Euclidean, Manhattan, and Cosine distance. We additionally tested various linkage types including complete link, average link, single link, and Ward's method. We found no difference between the clustering of counties using either the hierarchical clustering or the k-means analysis and thus we select k-means analysis due to computational efficiency.

In the case of the k-means analysis, we find that the silhouette method finds that best explanation where k equals 6 (Figure 6). However, the purpose of our clustering analysis is to visualize similar counties. We find that a k of six is not as visually effective (Figure 7) and thus choose a k of three to better visualize the counties that belong to each of the latent features.

3.5 Red-Green-Blue (RGB) Composite Map

The Red-Green-Blue (RGB) composite map (Figure 8) presents the linear combination of the components for each county where red is PC1, green is PC2, and blue is PC3. We predominantly observe red colors, which correspond to PC2, which corresponds to long-term reduced mobility at the west coast and the northeast coast. We can also observe a strong influence of green, corresponding to PC2, which corresponds to no reduction of mobility in the central United States. We also observe large patches of purple (red and blue) corresponding to a mixture of PC1 and PC3 in the midwest, New York, New Jersey, and other states, which corresponds to a mixture of long-term and short-term reduction of mobility having an absence of PC2.

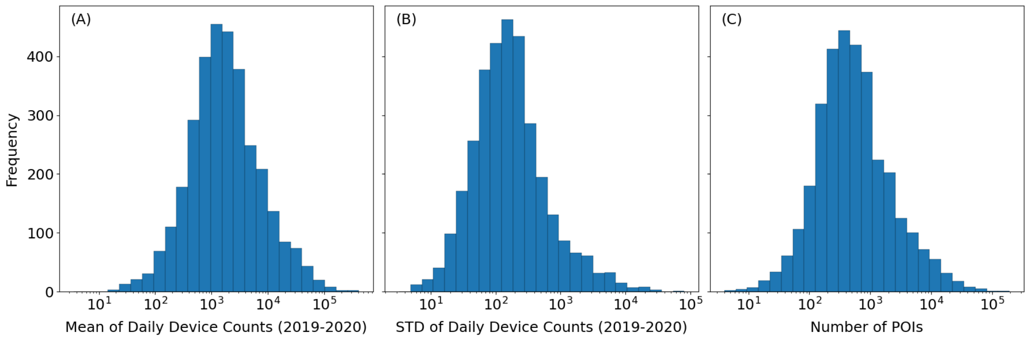

Figure 9: Geographic coverage of the data including (A) distribution of mean device counts per county, (B) distribution of sum of device counts per county, (C) distribution of standard deviation of device counts per county, and (D) distribution of sum of POIs per county.

3.6 Analysis of Geographic Coverage of the Data

To provide more information about the SafeGraph data that our study is based on we calculated descriptive statistics of device counts for each county per day. There is an average of 5433.76, a max of 414085.93, a min of 8.47 devices per U.S. county per day, Figure 9A shows the distribution of the mean number of observed devices (average over the $365 * 2 = 730$ days used in our study) across all 3100 counties included in our study. We observe that most counties have thousands of observations per day, with a few counties only have tens of observations per days and other counties having hundreds of thousands of observations per day. Figure 9A also shows the distribution of standard deviation of device counts (over 730 days) of all counties. To understand the spatial distribution of POIs among counties in the SafeGraph Places dataset we used a spatial join to count the total number of POIs in each county, as shown in Figure 9C. There is an average of 1788.84, max of 195232, min of 3, and standard deviation of 5941.88 POIs across counties. We found that geographic coverage at the county level is 100% for both device counts and POIs, meaning no counties were excluded due to lack of data.

References

[1] Thorndike RL. Who belongs in the family? Psychometrika. 1953;18(4):267–276.

[2] MacKay DJC. Information Theory, Inference & Learning Algorithms. Cambridge University Press; 2002.

[3] Rousseeuw PJ. Silhouettes: a graphical aid to the interpretation and validation of cluster analysis. Journal of computational and applied mathematics. 1987;20:53–65. doi:10.1016/0377-0427(87)90125-7.
