# Supplementary material for: Change of human mobility during COVID-19: A United States case study
Source: PLoS One. 2021 Nov 2;16(11):e0259031. doi: 10.1371/journal.pone.0259031 (PMC8562789; doi:10.1371/journal.pone.0259031)
Supplement: Attachment — Submitted filename: PONE-D-21-10041 Response to Reviewer and Editor.pdf [file pone.0259031.s002.pdf]

Manuscript ID PONE-D-21-10041

Change of human mobility during COVID-19: A United States case study

PLOSONE

We are grateful to the Editor and to the anonymous reviewer for their feedback. We have addressed all comments and indicated where in the manuscript the changes have been made, marked in blue and red font.

Our responses to the comments are as follows:

Response to Editor Comments:

1. Please ensure that your manuscript meets PLOS ONE's style requirements, including those for file naming. The PLOS ONE style templates can be found at

https://journals.plos.org/plosone/s/file?id=wjVg/PLOSOne_formatting_sample_main_body.pdf and

https://journals.plos.org/plosone/s/file?id=ba62/PLOSOne_formatting_sample_title_authors_affiliations.pdf

The manuscript meets PLOS ONE's style requirements.

2. Thank you for stating the following in the Acknowledgments Section of your manuscript:

This research is supported by National Science Foundation "RAPID: An Ensemble Approach to Combine Predictions from COVID-19 Simulations" grant DEB-2030685.We note that you have provided funding information that is not currently declared in your Funding Statement. However, funding information should not appear in the Acknowledgments section or other areas of your manuscript. We will only publish funding information present in the Funding Statement section of the online submission form.

Please remove any funding-related text from the manuscript and let us know how you would like to update your Funding Statement. Currently, your Funding Statement reads as follows:

T.A. A.Z. H.K.

Award no: 2030685

Funder: National Science Foundation

Funder website: nsf.gov

The funders had no role in study design, data collection and analysis, decision to publish, or preparation of the manuscript.

Please include your amended statements within your cover letter; we will change the online submission form on your behalf.

There are two awards that should be acknowledged. Please update the funding statement as follows:

Award 1:

T.A. A.Z. H.K.

Award no: 2030685

Funder: National Science Foundation

Funder website: nsf.gov

The funders had no role in study design, data collection and analysis, decision to publish, or preparation of the manuscript.

Award 2:

T.A. A.Z. H.K.

Award no: 2109647

Funder: National Science Foundation

Funder website: nsf.gov

The funders had no role in study design, data collection and analysis, decision to publish, or preparation of the manuscript.

3. We note that Figures 5, 6, 7, 9, 10 in your submission contain map images which may be copyrighted. All PLOS content is published under the Creative Commons Attribution License (CC BY 4.0), which means that the manuscript, images, and Supporting Information files will be freely available online, and any third party is permitted to access, download, copy, distribute, and use these materials in any way, even commercially, with proper attribution. For these reasons, we cannot publish previously copyrighted maps or satellite images created using proprietary data, such as Google software (Google Maps, Street View, and Earth). For more information, see our copyright guidelines: http://journals.plos.org/plosone/s/licenses-and-copyright.

We can confirm that the figures that contain map images do not contain copyrighted material.

4. Please review your reference list to ensure that it is complete and correct. If you have cited papers that have been retracted, please include the rationale for doing so in the manuscript text, or remove these references and replace them with relevant current references. Any changes to the reference list should be mentioned in the rebuttal letter that accompanies your revised manuscript. If you need to cite a retracted article, indicate the article's retracted status in the References list and also include a citation and full reference for the retraction notice.

We have ensured that the reference list is complete and correct.

Response to Reviewers Comments

1. Is the manuscript technically sound, and does the data support the conclusions?

The manuscript must describe a technically sound piece of scientific research with data that supports the conclusions. Experiments must have been conducted rigorously, with appropriate controls, replication, and sample sizes. The conclusions must be drawn appropriately based on the data presented.

Reviewer #1: Yes

---

2. Has the statistical analysis been performed appropriately and rigorously?

Reviewer #1: Yes

---

3. Have the authors made all data underlying the findings in their manuscript fully available?

The PLOS Data policy requires authors to make all data underlying the findings described in their manuscript fully available without restriction, with rare exception (please refer to the Data Availability Statement in the manuscript PDF file). The data should be provided as part of the manuscript or its supporting information, or deposited to a public repository. For example, in addition to summary statistics, the data points behind means, medians and variance measures should be available. If there are restrictions on publicly sharing data—e.g. participant privacy or use of data from a third party—those must be specified.

Reviewer #1: Yes

---

4. Is the manuscript presented in an intelligible fashion and written in standard English?

PLOS ONE does not copyedit accepted manuscripts, so the language in submitted articles must be clear, correct, and unambiguous. Any typographical or grammatical errors should be corrected at revision, so please note any specific errors here.

Reviewer #1: No

We have revised the manuscript and corrected for any typographical and grammatical errors.

---

5. Review Comments to the Author

Reviewer #1: This is a well-constructed and useful paper. The authors present a robust methodology, which is essentially a large scale clustering project, and with which I have no quibbles. My comments pertain more to reconciliation of data science vs. epidemiology terminology, and the need to further caveat the data sources.

Thank you for your feedback. Please note we have itemized each comment 5.1., 5.2., and so on to help in the organization of our responses.

5.1. Abstract: "Mobility in 2020 for US counties can be explained as a combination of three trends". It might be helpful to state these in the abstract.

We have added a brief description of the three trends in the abstract.

5.2. Table 1: This is excellent and useful. I have made a nearly identical table in my own research, so glad to see this find its way into print.

Thank you very much.

5.3. Methods section 2: "Delta Measure of Public Exposure"

This is semantic: I understand the meaning of this within this context. However, as an epidemiologist, the term "exposure" has a more specific meaning, which is not adequately represented by gross mobility measures. Within epidemiology, "exposure" means direct exposure to the causative agent. Mobility is a proxy for that. The authors may consider modifying the term to avoid detracting from what it otherwise and excellent paper. Similarly, in the discussion "Long term exposure reduction" will likely raise the hackles of public health audiences. Consider "Long term mobility restriction" as a more accurate representation of what was measured.

Thank you for this feedback. We have renamed the Measure of Public Exposure (MoPE) and ΔMoPE to Time Spent in Public Places (TSPP) and ΔTSPP to more accurately reflect what was measured. We consider an increase in ΔTSPP as one proxy for increase in mobility, thus increasing risk of exposure. We have revised all parts of the paper to consistently reflect this change.

5.4. Discussion: While the data providers have gone to unprecedented lengths to make these data public during the pandemic, as the authors note, the descriptions of methods are sparse.

We have added additional details in Section 2.1., page 4 regarding SafeGraph's methods for data collection. We have added a discussion regarding Safegraph's sparse documentation for their methods for collecting and processing the data in Section 4, page 12. See also our response to comment 5.6 and 5.7.

5.5. In addition, there are few safeguards that the data will continue to be available, or any real measures of completeness. As such, more in the discussion is warranted about the potential limitations scientific reliance on corporate free data.

As part of the SafeGraph Data for Academics, academic researchers have no-cost access to SafeGraph data for non-commercial work. We agree that there are no safeguards, since changes to data access may occur beyond our control. For example, Safegraph recently stopped updating the Social Distancing Metrics dataset. However, the archived data is still available for academic researchers so that the **median_non_home_dwell_time** can be obtained from 01/01/2019 through 12/31/2020, ensuring the reproducibility of this study. We have included step-by-step instructions and the Python scripts for obtaining and transforming the data from Safegraph's **median_non_home_dwell_time** to the ΔTSPP on our github (https://github.com/GMU-GGS-NSF-ABM-Research/Mobility-Trends). We have added these details in Section 2.1., page 4 and Section 4, page 13.

To overcome the potential issues with changes to data access, we have made our derived data, ΔTSPP (for each county and each day), fully available on the GitHub repository. We have clarified this in the Supplemental Material, Section 2.

We have added a discussion on data access and elaborated on the issues of relying on corporate data.

5.6. [Section] 2.2 "We note that this measure only includes public POIs that are captured among the 6.5 million POIs in the SafeGraph Core Places database."

This is a perfect example of my previous point. We have no idea how dynamic or complete these POI are. Granted SafeGraph has a more transparent and vibrant community of practice than most of the other providers. But still, the reliance on proprietary data has limitations that could be more clearly addressed.

We have added additional details in Section 4, page 12 and 13 to quantify the completeness and bias of the data. See also our response to comment 5.4 and 5.7.

5.7. Methods: Please describe geographic missingness in the dataset, e.g., arising from fewer POI or lower cell trace volume, or measurement uncertainty. What percent of US counties are covered?

We have performed additional analysis to quantify the geographic completeness of the data. We have added these details to the Discussion on Section 4, page 12 and 13 and Supplemental Materials. Specifically, we had added Figure 2 to the Supplementary Material to See also our response to comment 5.4 and 5.6.

5.8. "To reduce the dimensionality of R we truncate the SVD to obtain only the first K dimensions "

Please define kappa a little better to give a real world sense of the truncation.

In PCA, k is the number of latent features we wish to retain, where in our study k = 3 (PC1, PC2, PC3). There was a gap in the text where we switched abruptly from k to 3 without explaining. We have clarified this in the text in Section 2.3, page 5.

5.9. "SVD assumes that the ∆MoPE is derived from the sum of latent (individual) mobility changes."

Is it sum or averaged? The mobility measures usually report mean changes, so there is an additional assumption there.

We have corrected this on Section 2.3, page 5. SVD assumes that the true mobility is the linear combination of latent features which represent mobility change.

5.10. Methods: How are the 3 latent PCs measured/differentiated in the aggregate mobility data? For example, how are health professionals identified?

We make assumptions based on each latent feature or mobility pattern about the individuals that might contribute to these patterns. We have clarified this in Section 3.2.1., page 8.

5.11. Methods: Can you provide more information on SafeGraph "footfall" metric? Does this correspond more in urban areas where foot traffic is more common?

SafeGraph identifies when a device visits a POI using a visit attribution algorithm (https://www.safegraph.com/blog/revealing-safegraphs-secret-method-for-getting-accurate-store-visits-from-gps-data). Foot traffic or footfall in this respect refers to setting foot in a POI rather than walking on foot to a POI.

5.12. Methods: "it was discovered that the only neighboring county to Fairfax City in Fairfax County,"

Virginia is an annoying case in county-city geography, in my experience. Were both city and county designations used?

Anything that has a FIPs code attached to it (counties, cities, and boroughs) is designated a county. Thus, Fairfax City is identified at the county level. This has been clarified in the text in Section 2, page 3.

5.13. Methods: "Pearson's R coefficient "

This is a measure of linear association. Is there any a priori reason to believe the relationship to be linear?

Our hypothesis is that there is a linear relationship between the strength of the PCs in explaining county mobility and different county variables. We did not investigate non-linear relationships. We clarify this in Section 2.6, page 6.

5.14. Methods: "variety of explanatory variables, including income, political leaning, employment, and COVID-19 cases and deaths for each county "

Is this the complete list of variables? COVID cases and deaths were not consistently reported during the study period. For example, were antibody and rapid test positives included? Was repeat testing for the same individual accounted for? Despite being widely reported in data science and news media, the epidemiologic veracity of case counts is highly questionable. This should be caveated accordingly. Other journals have refused to publish results based solely on these numbers.

The complete list of variables can be found in Table 3. We have acknowledged the caveat of using COVID-19 cases and deaths in Section 3.5, page 11.

5.15. Figure 3 vs. 4 -- Suggest a different color scheme between graphs because causal readers may confuse the two legends since the lines are so similar.

We have changed the colors of Figure 3.

5.16. Figure 6: I do not understand the color wheel and the colored lines extended from it?

We have corrected Figure 6 so that the color wheel and the colored lines match.

5.17. I am curious why baseline commute times/distances, weather, and stay-at-home orders were not considered as explanatory variables?

Thank you. We will consider this in future work. We have added this to Section 4, page 13.

5.18. The figure resolution and map projection was of low quality in the review PDF. I assume this will be corrected in the final version.

We confirm that we have indeed submitted high resolution figures.
