# Supplementary material for: Change of human mobility during COVID-19: A United States case study
Source: PLoS One. 2021 Nov 2;16(11):e0259031. doi: 10.1371/journal.pone.0259031 (PMC8562789; doi:10.1371/journal.pone.0259031)
Supplement: Attachment — Submitted filename: PONE-D-21-10041R1 Response to Reviewers.pdf [file pone.0259031.s003.pdf]

Manuscript ID PONE-D-21-10041R1

Change of human mobility during COVID-19: A United States case study

PLOSONE

We are grateful to the Editor and to the two anonymous reviewers for their feedback. We have addressed all comments and indicated where in the manuscript the changes have been made, marked in blue and red font.

Our responses to the comments are as follows:

Reviewer #1

The edits and revision look good. Thanks for the attention to detail! The changes, both semantic and substantive are adequate. This is a nice paper. I look forward to being able to cite it.

Thank you.

Reviewer #2

I have read the manuscript “Change of human mobility during COVID-19: A United States case study”. The manuscript suggest a novel approach to identify the changes in human mobility between the years 2019 and 2020, using SafeGraph measure of median non home dwell time for each US county. The authors estimate the daily change in the measure between 2019 and 2020 in each county, find the principal components that explain most of the variance in the received time series, cluster the different county according to their principal components, conduct a spatial autocorrelation analysis between the counties, and a general correlation analysis of socio-demographic variables. The manuscript is concise, clear, and presents a solid framework for analyzing the change in mobility across the US due to the COVID-19 pandemic. However, the manuscript includes major pitfalls that should be addressed to in order to be published.

I divide my comments into general comments and specific comments.

General

1. In figure 2, it seems that TSPP in the summer of 2019 is lower than TSPP in January 2020. Although the authors do relate to the issue of the seasonal anomalies in the winters of both years, it seems highly unlikely that TSPP is higher in January of 2020 than in the summer of 2019. I suggest the authors run another check over the calculation to observe any mistakes. However, if no mistakes are found, I suggest the authors provide accurate data regarding the weather in each county and show that the weather in each county was indeed extreme. Since county s are not equal in size, a bias towards more dense areas exists, and extreme weather conditions in one of the metropolitan

areas can affect the results. However, due to the fact that no data from previous years exist, and 2019 is presumed to be a baseline year, it is crucial to validate the weather conditions(for example, by using NOAA's GHCN data) against the TSPP in order to explain the anomalies in TSPP.

Based on NOAA's GHCN data, it is clear that the US as a whole experienced a mild winter season (January - April 2020) with warmer average temperatures and roughly 50% less snow on the ground than in comparison to the 2019 winter season. This, in combination with panic buying behavior at the beginning of the pandemic, helps to explain the high mobility in January, February, and early March. We have updated the text and added Figure 3 (snow depth) and 4 (average temperature) to Section 3.1 of the Supplemental Materials to present these findings. Any further analysis to explain the mobility data based on weather conditions goes beyond the objective and scope of the study.

2. Regarding the principle components analysis – what led the decision to choose only 3 PCs? It is important to show the distribution of the contribution of the PCs to the variability, and then explain what led to the decision to choose only the first three.

The explained variance for each PC is presented in Figure 5 and Table 1 of Section 3.3. of the Supplemental Materials. We use the elbow method to determine that 3 PCs are sufficient.

3. The choice of k = 3 in k means clustering cannot be explained using the 3 significant PCs. I suggest the author find a better explanation for using k =3, such as the k means bend method or other methods that exist for hierarchical clustering.

We use the k-means clustering as a visualization tool so that we can observe which counties have a similar weighted combination of the three PCs. Thus, we arbitrarily choose a value of k = 3. Although we find that the silhouette method finds a better explanation where k = 6 (see Figure 6 in Section 3.4 of the Supplementary Materials), it is not as effective visually (see Figure 7 in the Supplementary Materials).

Specific

1. In definition 1, j should run between -3 and 3, in order to should that the moving average is of the middle day of the 7 day average

The reviewer is correct. In our implementation we indeed use a centered moving average of seven days. Definition 1 has been corrected to for the index j to run between -3 and 3, and we have clarified that the defined TSPP of a time series is only defined between the fourth and fourth-to-last element of the time series.

2. In definition 2, it should be noted that delta-TSPP is a by county measure, and therefore it should be parametrized and indexed as well.

The parameter R denotes the spatial region, such as a county. This was already parameterized in the previous manuscript. No changes were made to Definition 2.

3. In figure 2, an error bar/boxplot should be added for each day, in order to get a sense of the distribution of TSPP in the different counties.’

We have added Figure 1 and Figure 2, presenting the boxplots for each week to the Supplementary Materials.

4. 2.5 page 6: what state is Fairfax county?

We have updated the text with this information to clarify that Fairfax County is part of Virginia.

5. Figure 6 is unreadable. I would remove it from the paper as it confuses the reader, and it is somewhat redundant given figure 5.

We have moved this figure to Section 3.5 of the Supplemental Materials.

6. I suggest adding the share of population over 60 as one of the correlation analysis socio-demographic variables – it can help explain the positive coefficient of deaths per thousand in PC1, which is counterintuitive.

We have added the percent population over 65 as one of the correlation variables.
